# Sustained biologic-free and drug-free remission in rheumatoid arthritis, where are we now?

**DOI:** 10.1186/s13075-015-0707-1

**Published:** 2015-08-03

**Authors:** György Nagy, Ronald F van Vollenhoven

**Affiliations:** Department of Rheumatology, Semmelweis University, Faculty of Medicine, Semmelweis University, Budapest, 1023 Hungary; Department of Genetics, Cell and Immunobiology, Faculty of Medicine, Semmelweis University, Budapest, 1089 Hungary; Unit for Clinical Therapy Research, Inflammatory Diseases (ClinTRID), The Karolinska Institute, Stockholm, 17176 Sweden

## Abstract

The advent of new medications and new treatment strategies for rheumatoid arthritis has made it possible to achieve remission in more patients than before. Furthermore, recent clinical trials and register studies suggest that some patients who initially required aggressive therapy may achieve biologic-free remission or even the ultimate goal of therapy, drug-free remission, resembling recovery. Here, we present a discursive review of the most important studies addressing these issues. Based on the overall results, it remains unclear if achieving biologic-free and drug-free remissions are primarily due to the natural course of the disease or to the early therapeutic intervention according to the ‘window of opportunity’ hypothesis. Although medication-free remission is only achievable in a small subset of patients, characterizing this patient cohort may provide important information about beneficial prognostic factors and the underlying mechanisms. In summary, in a subset of patients biologic-free and even drug-free remission can be achieved; pursuing these possibilities in practice may decrease the risk for long-term side effects and attenuate the economic burden of the disease.

## Background

**Fig. 1:**
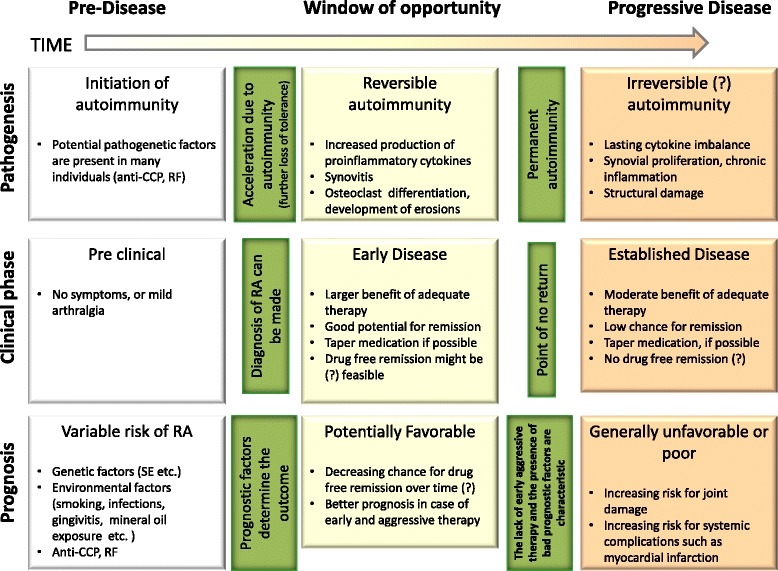
Phases of rheumatoid arthritis - a hypothetical model. Question marks indicate hypothetical statements. CCP, cyclic citrullinated peptide; RA, rheumatoid arthritis; RF, rheumatoid factor; SE, shared epitope

Early control of inflammation in rheumatoid arthritis (RA) generally results in better outcomes; thus, the current treatment strategy is to initiate aggressive therapy as soon as possible after diagnosis has been established, and to extend the therapy, according to the disease activity, to reach clinical remission [1] (Fig. 1). Currently we have many synthetic and biological disease modifying antirheumatic drugs (sDMARD and bDMARD, respectively) to achieve this aim. With appropriate use of biologicals and tight control strategies, remarkable improvement is achievable in clinical outcomes, and remission is possible in an increasing numbers of patients. Lifelong treatment with bDMARDs is very expensive and entails potential long-term side effects. According to the EULAR recommendations [2], if a patient is in remission, bDMARD and in the case of sustained remission even sDMARD therapy might be tapered. Here we summarize the current understanding of biologic-free and drug-free remission in RA.

Paradoxically, we know much more about optimal initiation than about optimal termination of pharmacotherapy in RA [3]. Sustained clinical remission is achieved in 20 to 40 % of patients, and generally requires ongoing therapy, but bDMARDs and/or sDMARDs may be able to be tapered for some of this patient group. A small but not inconsiderable proportion of patients with RA may have a chance for drug-free remission, which might be due to the natural course of the disease and/or to the therapies used; the key question is whether currently available medications may alter this chance [4].

In addition to genetic risk factors, such as PTPN22 and HLA-DRB1, environmental factors also have a central role in the pathogenesis of RA [5]. Cigarette smoking is the best established environmental risk [6], while moderate alcohol consumption seems to be protective. The production of anti-citrullinated protein antibodies (ACPAs) and rheumatoid factor (RF) before the onset of the disease has been shown and these are associated with initiation of autoimmunity and loss of tolerance towards self structures [7]. Genetic and environmental factors together with unknown triggers (infections and so on) may directly lead to initiation of RA. Regulatory T helper cells appear to have a role in peripheral tolerance, and impaired function of these cells may contribute to a loss of immune tolerance in RA [8]. The activation of both the adaptive and innate immune systems is characteristic; due to accelerated osteoclast differentiation and activation, bone erosions develop in the first months after the onset of the disease and are generally associated with sustained inflammation. RA is characterized by an increased risk of cardiovascular disease, such as cerebrovascular events, heart failure, myocardial infarction, and shorter life expectancy than the general population [9].

It is widely accepted that cytokine production by many cell populations of the inflamed synovium has a central role in the pathogenesis of RA. Furthermore, several lines of evidence support that a complex network of proinflammatory cytokines and numerous positive feedback loops perpetuate the chronic inflammation. Biologicals may substantially remodel the network of cytokines, leading to significant clinical improvement [10]. Interleukin (IL)-1, IL-6, IL-17, IL-18, and IL-33 and tumor necrosis factor (TNF) are overexpressed in RA. While TNF and IL-6 blockade are effective treatment options, the effectiveness of IL-1 inhibition is rather modest. According to the available data, the effect of the IL-17 blocker secukinumab appears to be also moderate [11, 12]. The possible reason for the variation in the clinical benefit of cytokine blocking-based therapeutic approaches is that cytokine-driven inflammation [13] might be distinct between individual RA patients.

## Remission induction and tapering biologicals

According to the concept of the ‘window of opportunity’ [14–16]**,** aggressive treatment in the early phase of the disease may lead to excellent improvement and sustained benefit (Fig. 1). It is hoped that remission induction in the window of opportunity allows medications to be tapered in the medium term to maintenance therapy. This concept is supported by the observation that therapeutic response in the first 3 months of therapy predicts the potential of reaching remission later [17]. A potential explanation for the effectiveness of early intervention is that autoimmunity might not be fully established during this phase. Several studies have investigated tapering biologicals.

Quinn et al. [18] described for the first time sustained responses after the termination of a biological therapy. Patients with early RA (<12 months of symptoms, 65 % RF-positive) were treated with methotrexate (MTX), and were randomized to be treated with infliximab or placebo for 12 months. Patients were followed for an additional 12 months. The primary endpoint of this double blind study was synovitis, measured by magnetic resonance imaging (MRI). At 12 months the American College of Rheumatology (ACR)50 and ACR70 responses were better in the MTX plus infliximab group (*P* < 0.05 and *P* < 0.05, respectively), no new erosions were found, and the MRI scores were significantly better than in the MTX plus placebo group. More remarkably, 1 year after the discontinuation of infliximab, 70 % of patients still had sustained excellent clinical response, and the quality of life and functional advantages were still manifest, thereby supporting the window of opportunity hypothesis.

The Behandel-Strategieën (BeSt) study clearly showed that a treat-to-target approach provides sustained clinical and radiological benefit in RA [19, 20]. In this study patients were randomized to four remission induction treatment groups: group 1, sequential monotherapy (n = 126); group 2, step up combination therapy (n = 121); group 3, initial combination with prednisone (n = 133); group 4, initial combination with MTX and infliximab (n = 128). The maximum disease duration was 2 years. Disease Activity Score (DAS) was measured every 3 months, and medications were adjusted according to the actual disease activity. The goal in all treatment arms was to reduce disease activity as soon and as effectively as possible. DAS44 ≤ 2.4 was defined as good clinical response, and if DAS44 was >2.4, the next treatment step was applied. The medication was tapered to monotherapy if DAS44 was ≤2.4 for at least half a year.

In group 1 treatment was started with MTX 15 mg/week and in the case of insufficient response the dose of MTX was raised to 25 mg/week, then changed to the next medications: sulphasalazine (SSA); leflunomide; MTX plus infliximab with stepwise increases in infliximab dose from 3 mg/kg to 10 mg/kg; gold; MTX plus ciclosporin (CSA) plus prednisone; azathioprine (AZA) plus prednisone. In group 2, patients with inadequate response to MTX received the following treatments: MTX plus SSA; MTX plus SSA plus hydroxychloroquine (HCQ); MTX plus SSA plus HCQ plus prednisone; MTX plus infliximab with increasing infliximab doses as in group 1; MTX plus CSA plus prednisone; gold; AZA plus prednisone. In group 3 the order of treatments was: MTX plus SSA plus prednisone; MTX plus CSA plus prednisone; MTX plus infliximab with increasing infliximab doses as in group 1; leflunomide; gold; AZA plus prednisone. The order of treatments in group 4 was: MTX plus infliximab with increasing MTX doses from 15 to 25 mg/week, then with increasing infliximab doses as in group 1; SSA; leflunomide; MTX plus CSA plus prednisone; gold; AZA plus prednisone.

In group 4 43 % of patients were in remission (DAS44 < 1.6) and 56 % completed infliximab treatment during the first 2 years of the study [19]. In a *post hoc* analysis of all four groups, 45 % of patients could discontinue the biological therapy and 52 % of these patients did not restart during the 7.2 year median follow-up time [20]. Shared epitope (SE) positivity, smoking and prolonged infliximab treatment were associated with restarting infliximab; that is, with the inability to maintain remission without the anti-TNF agent. Conversely, SE negativity, non-smoking and shorter anti-TNF treatment were predictors for sustained biologic-free remission.

In the Infliximab as Induction therapy in Early Rheumatoid Arthritis (IDEA) study (a 78 week, double-blind, randomized, controlled study) remission induction with MTX plus infliximab and MTX plus high-dose intravenous steroid were compared in a treat-to-target approach [21]. This study included 112 DMARD-naïve patients with early RA (3 to 12 months symptom duration). All patients received at least 10 mg/week MTX, increased to 20 mg/week or to maximum tolerated dose. Patients were randomized to receive either 3 mg/kg infliximab according to the standard protocol, or 250 mg intravenous methylprednisone at week 0 and placebo infusions at weeks 2, 6, 14 and 22. From week 26, in the unblinded, open label phase of the study patients with an inadequate response (DAS44 > 2.4) in the infliximab group were treated with increased infliximab doses (up to 10 mg/kg), or biological therapy was stopped and MTX was changed to other sDMARDs. In the high dose steroid group, if DAS44 was >2.4, the following treatments were applied: MTX plus SSA plus HCQ; then MTX plus leflunomide; oral MTX was changed to subcutaneous MTX; CSA plus subcutaneous MTX; CSA plus subcutaneous MTX plus oral prednisone. Infliximab was stopped if DAS44 was <1.6 for 6 months. Overall there was no significant difference in the efficiency of the two therapeutic strategies; 24.5 % (14/55) of the patients discontinued infliximab therapy due to sustained remission, and 76 % (11/14) of those were still in remission at the end of the study.

The Japanese Remission Induction by Remicade in RA (RRR) study [22] was designed to investigate if infliximab (3 mg/kg) with concomitant MTX might be stopped in patients with persistent low disease activity (DAS28 < 3.2 for >24 weeks). The mean disease duration was 5.9 years and the mean DAS28 score was 5.5 at baseline. The primary endpoints were: 1, low disease activity after discontinuing infliximab at 1 year; 2, modified total Sharp score (mTSS) progression <0.5 at 1 year. Of the 102 patients rated at year 1, 55 % (56) were able to discontinue infliximab (RRR-achieved) and 44 (43 %) reached remission (DAS28 < 2.6). Within 1 year after stopping infliximab 29 patients had flared. The mTSS was comparable in the RRR-achieved and RRR-failed patient groups. Patients who were able to discontinue infliximab were younger, had lower mTSS and had shorter disease duration, further supporting the window of opportunity hypothesis.

In the recently published 78 week randomized, double blind Optimal Protocol for Methotrexate and Adalimumab Combination Therapy in early Rheumatoid Arthritis (OPTIMA) trial, MTX naïve patients with early RA (disease duration <1 year) were randomized to receive either adalimumab (40 mg biweekly) plus MTX or placebo plus MTX therapy for 26 weeks (period 1) [23]**.** After 26 weeks, patients who reached stable low disease activity (DAS28 < 3.2) were randomized either to continue or to stop adalimumab, and were followed for an additional 52 weeks (period 2). Forty-four percent of patients who were treated with adalimumab and MTX (as induction therapy) reached low disease activity. Importantly, the benefit was often maintained after cessation of adalimumab therapy: 82 of 101 patients had DAS28 < 3.2 at week 78, suggesting that MTX maintenance is effective in most patients following successful induction therapy.

The High Induction Therapy with Anti-Rheumatic Drugs (HIT HARD) study investigated the effect of induction therapy with MTX plus adalimumab [24]. Patients with early RA (average disease duration <1 year) were randomized 1:1 into MTX plus placebo and MTX plus adalimumab in this 48-week trial. After week 24 all patients continued MTX monotherapy. The primary outcome was DAS28 at week 48. At week 24 the remission rates were significantly higher in the adalimumab plus MTX group (*P* = 0.009). In the MTX plus adalimumab arm 45 % of patients were in remission (DAS28 < 2.6) at week 24, and about 90 % of those were still in remission at week 48, further supporting that MTX maintenance therapy is effective in a significant proportion of patients for sustaining remission after the cessation of biological therapy. However, there was no statistically significant difference between the two arms in clinical efficacy at week 48.

Initial MTX monotherapy (group 1) and the combination of MTX with adalimumab (group 2) in early RA (maximum disease duration 6 months) were compared in the prospective, unblinded, randomized, 1 year GUEPARD trial [25]. In case of insufficient response at week 12 in group 1, patients were treated with MTX plus adalimumab, MTX plus etanercept, or MTX plus leflunomide. In group 2, if the DAS28 was <3.2, adalimumab was stopped; 39 % of patients in group 2 had low disease activity from week 12 until the end of the study while receiving MTX monotherapy. There was no significant difference in clinical or radiological outcomes between the two groups. It is important to point out that both the HIT HARD and the GUEPARD trials may have been underpowered to detect the true long-term effects of early anti-TNF therapy.

## Drug-free remission

In addition to decreasing the signs and symptoms of RA, early and aggressive therapy may also improve the underlying immune disturbance, leading to drug-free remission in a small subset of patients. Drug-free remission has been described in several patient groups.

In the BeSt study, after the second year, if the DAS44 was <1.6 for at least half a year, the DMARD was tapered and discontinued. Thirteen percent of the patients were in medication-free remission after 4 years [26]. Male gender, lack of ACPA and short symptom duration were associated with drug-free remission. Furthermore, 48 % of patients were in remission and 14 % in drug-free remission after 5 years; 14 %, 16 %, 10 %, and 19 % of patients reached medication-free remission in groups 1 to 4 after 5 years [27]. It is uncertain if the treat-to-target strategy or the natural course of the disease (irrespective of the drugs used) had more influence on the successful tapering of the medications. Although the initial combination therapy with MTX and infliximab led numerically to the most favorable result in terms of achieving drug-free remission (19 %), further studies are needed to clarify if there is significant difference between the strategies in reaching this aim.

The prevalence of and predictive factors for medication-free remission were studied in the British Early Rheumatoid Arthritis Study (ERAS) cohort and in the Leiden Early Arthritis Clinic (EAC) cohort (prospective inception cohorts) [28]. All patients were treated with sDMARDs. Sustained medication-free remission (defined as no synovitis after terminating the DMARD therapy) was observed in 15 % of the patients in the EAC and 9.4 % in the ERAS cohort. Short symptom duration, SE-negativity, RF-negativity, acute onset, not smoking and minimal radiographic damage were associated with drug-free remission in both patient cohorts.

The chance for sustained drug-free remission was compared retrospectively in the case of DAS-driven and non-DAS-driven therapy [29]. Patients included in the BeSt study (a randomized treatment cohort) received DAS-driven therapy, while patients in the EAC cohort were treated in a non-DAS-driven way. The prevalence of medication-free remission was comparable in the DAS-driven (9.8 %) and non DAS-driven (10.6 %) cohorts. Although ACPA-positivity is associated with worse prognosis, ACPA-positive patients had a better chance for drug-free remission if they were treated in a DAS-driven way. The absence of RF and ACPA, SE-negativity, male gender, lower health assessment questionnaire (HAQ) score and DAS at baseline were associated with sustained drug-free remission in the DAS-guided cohort [29]. In both cohorts short symptom duration and lack of ACPA were independent prognostic factors for drug-free remission.

The Etanercept and Methotrexate in Patients to Induce Remission in early Arthritis (EMPIRE) trial compared the efficacy of etanercept plus MTX with MTX monotherapy for remission induction [30]. DMARD-naïve patients with early inflammatory arthritis (less than 3 months disease duration) and either RF-, ACPA- or SE-positivity were included in this 78-week randomized superiority trial. The primary endpoint was no tender or swollen joint at week 52. Patients received placebo or etanercept injections until they had no tender and swollen joint for 26 weeks, and at week 52 all patients stopped receiving injections. After the cessation of etanercept or placebo, if patients were in remission for at least 12 weeks, MTX was discontinued. There was no difference between the patient groups in achieving the primary endpoint. In both groups 3.6 % of patients reached sustained drug-free remission at week 78, suggesting that early treatment with etanercept does not increase the chance for drug-free remission.

In the PRIZE study, patients with early RA who reached remission (DAS28 < 2.6) following treatment with 50 mg etanercept plus MTX for 52 weeks were randomized to 25 mg etanercept plus MTX, MTX monotherapy, or placebo arms for 39 weeks [31]**,** then all medications were stopped and the patients were monitored for an additional 26 weeks. At week 117, after treatment withdrawal, 42 %, 30 % and 22 % in the etanercept plus MTX, MTX monotherapy, and placebo arms, respectively, were still in remission. This means that 22 % of patients who were in remission with 50 mg etanercept and MTX were still in remission more than 1 year after the cessation of both drugs.

In the Assessing Very Early Rheumatoid arthritis Treatment (AVERT) trial the efficacy and safety of abatacept were studied in ACPA-positive patients with early (active synovitis for ≥8 weeks) RA [32]. Patients were randomized to 12 months abatacept plus MTX treatment, or to abatacept monotherapy or MTX monotherapy arms. Patients with DAS28 < 3.2 at month 12 entered another 12-month period with no treatment: 14.8 %, 12.4 % and 7.8 % of patients in the abatacept plus MTX, abatacept monotherapy and MTX monotherapy arms, respectively, were in remission (DAS28 < 2.6) at both 12 and 18 months. A significantly higher percentage of patients in the abatacept plus MTX arm were in remission at both 12 and 18 months compared with the MTX arm (*P* = 0.01 and *P* = 0.045, respectively), but there was no meaningful difference between the abatacept monotherapy and MTX arms at either time point. Drug-free remission was associated with lower baseline symptom duration, lower initial DAS28 and lower HAQ score.

The 3-year double-blind ACT-RAY study was designed to investigate the efficacy of tocilizumab plus MTX in RA using a treat-to-target strategy [33]. Patients who were in sustained remission discontinued tocilizumab, and if remission was maintained, sDMARDs were stopped as well: 50.4 % of patients stopped tocilizumab and eventually 5.9 % achieved drug-free remission. Although the majority of patients who quit the bDMARD flared, reintroducing tocilizumab led to clinical improvement in most cases.

The prevalence of drug-free remission among patients treated with tocilizumab monotherapy (DREAM study) was also investigated recently [34]. Ten percent of patients were able to discontinue tocilizumab therapy. Low matrix metalloproteinase 3 and low serum IL-6 levels were predictors of low disease activity.

Some limitations of the studies discussed here should be acknowledged. Complete molecular remission may not happen in all patients who are in clinical remission, and therefore some radiographic progression may occur even during clinical remission. It is possible that some of the patients who could completely stop all medications and had sustained remission did not have RA. Although the 1987 classification criteria [35] were used in the majority of these studies (except the EMPIRE and the AVERT trials; all patients in EMPIRE had early inflammatory arthritis and not all fulfilled the 1987 classification criteria [30]; in the AVERT trial, patients were described as early RA [32]), some misclassification may have occurred. However, the similar rates of medication-free remission in the different patient populations (different cohorts, different serological features), chronic polyarthritis and the structural joint damage described for most patients argue against misclassification in these studies.

## Translating pathogenesis into clinical phases, a hypothetical model

Our hypothesis is that, in accordance with the window of opportunity theory, the potential reversibility of autoimmunity decreases over time in RA and this alters the potential efficacy of therapies. Based on our hypothesis and the features of the different phases of RA, pre-disease phase individuals with ACPA-, RF- and SE-positivity have a significant risk for RA, especially if they have arthralgia (Fig. 1) and the risk is further increased in smokers. This phase, when autoimmunity is already present, can be transformed into definitive RA, the window of opportunity phase, which is associated with the acceleration of autoimmunity, further loss of tolerance in molecular level and unequivocal clinical symptoms. This phase is characterized by increased production of proinflammatory cytokines and consequential synovitis, osteoclast activation and development of erosions. In the early phase of the disease aggressive therapy leads to disproportionate benefits and patients have a good chance for remission, and even drug-free remission might be feasible. Importantly, autoimmunity appears to be reversible in this phase in some patients. Since autoimmunity is characterized by the loss of tolerance toward self-structures [7], drug-free remission at the molecular level probably means not only complete suppression of disease activity, but also the re-establishment of tolerance. Improved function of regulatory T helper cells is likely necessary for the recovery of normal immunological tolerance. Later, in the progressive phase of the disease, autoimmunity is no longer reversible, and chronic synovitis and lasting cytokine imbalance lead to further structural damage. In this late phase adequate therapy leads to moderate benefit, patients have a low chance of remission and there is no chance for drug-free remission.

In all probability, different treatment strategies with distinct effects on the cytokine cascades specify the chance of medication-free remission. This model suggests that, among other known and unknown factors, disease duration is a crucial risk factor for progressive disease, which is generally characterized by irreversible autoimmunity.

## Conclusions

Available clinical trials have demonstrated that appropriate therapy, initiated during the window of opportunity, may lead to rapid and sustained improvement in RA, frequently enabling the cessation of biologicals and in some cases all medications might be tapered. Although only a few trials aimed to study drug-free remission, there is some evidence that biologicals increase its likelihood; there might be differences between medications in this respect. As it seems the prevalence of medication-free remission is now around 3.6 to 22 %, further studies are needed to select the most favorable treatment strategies. The possible roles of disease duration, environmental and genetic factors should be investigated as well. It is crucial to identify those patients who have a realistic chance to taper medications; reliable biomarkers are needed to reach this aim. It is especially important to understand the conditions that lead to the re-establishment of tolerance during remission. Understanding the mechanism of drug-free remission at the molecular level might provide invaluable information to develop curative therapeutics in RA.

## Abbreviations

ACPA: Anti-citrullinated protein antibody
ACR: American college of rheumatology
AVERT: Assessing very early rheumatoid arthirits treatment
AZA: Azathioprine
bDMARD: biological disease modifying antirheumatic drug
BeSt: Behandel-Strategieën
CSA: Ciclosporin
DAS: Disease activity score
EAC: Early arthritis clinic
EMPIRE: Etanercept and methotrexate in patients to induce remission in early arthritis
ERAS: Early rheumatoid arthritis study
HAQ: Health assessment questionnaire
HCQ: Hydroxychloroquine
HIT HARD: High induction therapy with anti-rheumatic drugs
IL: Interleukin
MRI: Magnetic resonance imaging
mTSS: modified total Sharp score
MTX: Methotrexate
RA: Rheumatoid arthritis
RF: Rheumatoid factor
RRR: Remission Induction by Remicade in RA
sDMARD: synthetic disease modifying antirheumatic drug
SE: Shared epitope
SSA: Sulphasalazine
TNF: Tumor necrosis factor
